# Medical and Philosophical Intelligence

**Published:** 1815-01

**Authors:** 


### MEDICAL AND PHILOSOPHICAL INTELLIGENCE.

NATIONAL VACCINE ESTABLISHMENT.—At a Board holden on the 6th day of October, 1814, it was resolved,

That there be three classes of vaccinators, namely, *stationary*, *extraordinary*, and *corresponding*.

That the vaccinators of each class be members of the Royal College of Surgeons in London.

That every vaccinator retain his office during the pleasure of the Board; and upon such conditions as the Board shall, from time to time, judge proper.

That stationary and extraordinary vaccinators be chosen by the Board.

That corresponding vaccinators be appointed by the Board, or by the inhabitants of distant parishes or districts.

That the present stationary vaccinators be continued, upon the remunerative conditions under which they were chosen, until the Board shall otherwise determine.

That extraordinary vaccinators be resident within, or in the vicinity of, the metropolis; and that each of them be expected to sign a copy of the following form of engagement:

I hereby engage, as extraordinary vaccinator of the National Vaccine Establishment, to vaccinate, gratuitously, the infant-poor, and other proper objects, who shall apply, or be presented to me, for vaccination; to promote, to the utmost of my power, the practice of vaccination; and, upon every occasion, to act conformably to the instructions which I have received, or which I may receive, from the Board of the establishment.

That corresponding vaccinators be resident in the United Kingdom and its dependencies; not in the vicinity of the metropolis: and that, whether appointed by the inhabitants of parishes or districts, or by the board, each of them be expected to keep a register of the persons whom he shall vaccinate, with remarks upon interesting occurrences; and, annually, to communicate a summary thereof to the board of the establishment.

"That

That every corresponding vaccinator be allowed copies of the printed directions of the board relating to vaccination, and to supplies of vaccine lymph, upon due application to the board.

That a list of the members and officers of the board, and of the vaccinators under the three distinct heads, be annually printed; that a copy thereof be sent to each of such persons; and that copies be otherwise distributed, according to the directions of the board, for the public information and benefit.

That to induce the appointment of corresponding vaccinators, and, also, otherwise to further the designs of the establishment, an advertisement, in the following terms, be inserted in such public papers, and be otherwise printed and circulated, as the board shall from time to time direct; and that such advertisement have the signatures of the members of the board.

*National Vaccine Establishment.*—The Board of the National Vaccine Establishment, under the authority of government, in pursuance of their duty, to extend, by all possible means, the practice of vaccination, and thereby to accelerate the extermination of small-pox from this nation, as happily has already been effected in some countries where, before the introduction of vaccination, that disease was prevalent, and generally destructive; do recommend to the inhabitants of parishes in the United Kingdom and its dependencies, not in the vicinity of the metropolis, to appoint so many members of the Royal College of Surgeons in London as may be judged expedient, corresponding vaccinators of the National Vaccine Establishment; who shall each be expected to vaccinate, gratuitously, the infant-poor, and other proper objects, inhabitants of the district to which he shall be appointed; to keep a register of the persons whom he shall vaccinate, with remarks upon interesting occurrences; and annually to communicate a summary thereof to the board of the establishment, addressed to Dr. Hervey, National Vaccine Establishment, Leicester Square, under cover, to the Right Hon. the Secretary of State for the Home Department, Whitehall.

And every person who shall be appointed as herein is recommended, will be recognised by the board as a corresponding vaccinator of the National Vaccine Establishment; and will accordingly receive, free of expense, the printed directions of the board relating to vaccination; and at all times necessary supplies of vaccine lymph.

The board also earnestly solicit the enlightened and benevolent clergy of the United Kingdom and its dependencies, to exhort their parishioners favourably to receive, and justly to estimate, the blessing providentially pointed out for immediate security against the infection of small-pox, and eventually for the total eradication of that contagious, loathsome, and fatal disease; and particularly to impress upon their minds the special duty of the directors of institutions for the benefit of infant-poor, to obtain for the objects of their charitable intention timely and proper vaccination.

And the board solemnly call upon all persons, especially the magistrates magistrates of the United Kingdom, as they value the lives of human beings, to use their influence and authority in preventing the exposure of persons having upon them small-pox; and, so far, in arresting the propagation, and forwarding the extinction, of that pestilential malady.

| | |
|---|---|
| John Latham, M.D. President of the Royal College of Physicians. | William Blizard, Master of the Royal College of Surgeons. |
| Henry Ainslie, M.D. } Censors | Henry Cline, } Governors. |
| James Haworth, M.D. } Censors | William Norris, } Governors. |
| Thomas Hume, M.D. } Censors | |
| H. J. Cholmeley, M.D. } Censors | James Hervey, M.D. Registrar. |

---

At the meeting of the London Medical Society, on Monday, the 13th instant, Mr. STEVENSON mentioned that he had successfully performed Professor Assalini's operation for the artificial pupil. This was done in the presence of that gentleman. Mr. Stevenson has since had an opportunity of repeating it with equal success. He is convinced that this method exceeds all others hitherto proposed, especially where the opacity completely occupies the centre of the cornea.

---

The late MARTIN VANBUTCHELL, who made himself so remarkable, not to say ridiculous, by his long beard, his poney, and his embalmed rib, has rendered a greater service to the English public than is generally known. To him, it is said, we are indebted for the invention of elastic bracers. From the time that it has been the custom to be unnaturally short-waisted, the great difficulty has been to support the small-clothes. This was atchieved by tight waistband; the consequence of which was such a pressure on the parietes of the abdomen, and forced their contents through the ring. Frequent as hernias are, they are much less so in the rising generation since the introduction of bracers. For this remark we are indebted to Mr. Bell, who examines the boys brought to the Marine Society.

---

One of the French Journals mentions a circumstance which would argue a very imperfect state of civilization in some of their departments. In the town of Noyon, a man under Hydrophobia escaped from his nurses into the street, where he was actually shot by the populace, lest he should communicate the disease by his bite. The magistrates interfered in vain. We hope to lay before our readers more particulars of this extraordinary transaction.

---

The Medico-Chirurgical Society of Gaud, have proposed to give a prize medal, 300 franks in value, for the best dissertation on the following subject; the discussion on the merits of the respective memoirs will be made in the year 1815.—Question: What are the Diseases, internal or external, which from their appearance, symptoms, or situation, can be confounded with Syphilis; and what are the signs, phenomena, and the means by which they can be distinguished with certainty from that disease?—The memoirs written in Dutch, French, Latin, or English, must be sent, free of postage, before the close of July, 1815, to Mr. Kluyskens, perpetual secretary of the society. The memoirs should be accompanied with a sealed packet, containing the name and residence of the author.

---

*Congenital Hernia of the Heart;* by M. CHAUSSIER.—M. CHAUSSIER has presented to the Medical Society of Paris, the case of an infant who has a tumor at the superior and anterior part of the abdomen: it is soft, hemispherical, elevated about an inch from the surface of the body, two inches and a quarter broad, in which the alternate motions of the ventricles and auricles of the heart are evident.

The appearance and size of the tumor, occupying a space from the anterior and lower part of the thorax to the navel, changes with the movements of respiration. When the child inspires, the heart ascends, and in part seems to return into the thorax. In *expiration* the heart descends, and its pulsations are more conspicuous. The tumor sensibly increases in size and tension when it cries, or when it is put in an erect position, and *vice versâ.* The tumor diminishes when it is quiet, and the limbs are bent upon the trunk; a graduated pressure dissipated the tumor, but the respiration became more difficult; when the pressure was removed, the tumor returned.—The child sucks, performs all its functions, and is likely to live.

Senac, in his Traité du Cœur, lib. iv. chap. 8, relates a particular case where the heart had formed to itself a cavity in the diaphragm, which it forced downwards, and produced a strong pulsation in the epigastrium. Regis (Journ. des Scav. 1681,) relates that he dissected two little dogs, in which the heart was protruded from the thorax. Vaubonais (Acad. des Sciences, 1712,) speaks of a fœtus born at the eighth month of pregnancy, whose heart escaped from an opening at the upper part of the thorax. Martin Martinez, in a dissertation printed at Madrid in 1723, which Haller has inserted in the second volume of Disput. Anatom. relates a similar occurrence. Tourtelli (Journ. de Med. tome 62) saw a child where the heart escaped from the lower part of the thorax, and extended to about an inch above the umbilicus.

In all the cases where the heart has been exposed and deprived of its pericardium, death has occurred shortly after birth; not so when it is protected from the air and covered by the integuments.

In the military hospital of Dijon, I observed in a soldier, 27 years of age, of a robust constitution, the heart situated at the anterior part of the sternum, about the centre, covered only by the skin; the sternum was wanting at its lower part; the ribs which unite to the sternum were deprived of the cartilage by which they are connected with it. Notwithstanding this singular congenital conformation, this man's health was good. He had been many years a soldier, and had endured all the fatigues incident to his his profession without any untoward symptom. He was sent to the hospital for a slight indisposition, from which he soon recovered. M. Ramel (Journ. de Medicine, tome 49) relates a case very similar to that which is the subject of my observation. A girl, 10 years of age, was observed to have continual pulsations in the epigastric region, and experienced great uneasiness from the slightest pressure on this part. He recognized through the thickness of the integuments, that the heart was situated beneath the diaphragm, and by means of stays was enabled to remove all the inconveniences of her situation. Our deceased colleague at the Maternité Baudeloque, found in a child, that died soon after birth, two hearts, one in the thorax the other in the abdomen, which were united, and communicated with each other by different tubes, ramifications, and vascularies. In 1812, I saw at the Maternité a child much deformed in several parts. At the basis of the umbilical cord was found a herniary tumor, which not only contained the greater part of the abdominal viscera, but also the heart. An account of this is preserved in the second number of the Bulletin of the Society, published in 1813.—*Bulletin de la Faculté de Medicine de Paris.*

---

*Case of a Man whose Skin naturally white became black;* by M. Chomel, D.M.—Marin Charles Marion, 67 years of age, received into the Hospital la Charité for a pulmonary catarrh, added to an almost entire black discoloration of the skin. This man enjoyed tolerable health, with the exception of epistaxis and frequent pains of the head. In April last he began to perceive that his legs and arms assumed a blackish tint, but so indifferent was he about it, that he did not examine the trunk to discover if it was of the same colour. About this time there came languor and cough, his appetite failed, digestion was bad, his strength diminished; the body became more sensible to the impression of cold, and his legs were a little swoln towards the evening. A month after this he was admitted into the hospital, when the following appearances presented themselves:—The skin of the trunk, in all points of its extent, was of a black colour, but darker in some parts than in others; the lateral parts of the thorax and abdomen were the blackest, resembling the skin of the negro. The limbs were not so black as the trunk, and became toward the extremities gradually of a lighter tint; the skin was shining and remarkably soft; it did not exhale the odour of negroes, nor was it unctuous to the feel; it was dusted with a whitish powder from the natural desquammation of the epidermis, and especially from the action of scratching.

Nothing very remarkable was observed as to his general health; the expression of the figure was natural, appetite and digestion pretty good, respiration somewhat oppressed; the sleep was good, the legs and thighs œdematous, but it required strong pressure to produce depression, as in anasarca.

Interrogated as to the probable cause of this discoloration, the patient ascribed it to vermin and uncleanliness; he had been lousy, and had worn the same shirt a long time; he never had the itch, venereal disease, or any cutaneous eruption. In fifteen days after he came into the hospital, the œdema disappeared; but about the beginning of July new weakness came on, which increased so that the patient could not leave his bed. The difficulty of breathing increased, and the expectoration tinged with blood. The pulse was accelerated, and the œdema appeared in the inside of the thighs: on the 8th he died. On dissection the lungs were found suffused with blood. On cutting into the skin at several parts, the discoloration was found to arise from a layer of black matter on the surface; the epidermis was found connected to it, and could not by any means be separated from it.—*Bulletin de la Faculté de Medicine.*

---

The Edinburgh Reviewers, by their unfair treatment of Mr. Abernethy, have given Dr. Adams an opportunity of explaining, in an eighteen-penny pamphlet, the supposed complicated doctrine of his favourite master on the Vitality of the Blood.

---

Mr. Pettigrew has in preparation a new edition of Dr. Lettsom's Naturalists and Travellers' Companion, in which it is proposed to add an account of all the recent discoveries in the several branches of science therein treated of, which will be executed under the author's inspection, and will most probably appear early in the spring.

---

Dr. Bateman has published a third edition of his Synopsis. We have not yet had leisure to examine what alteration he has made in consequence of the case of Elephantiasis now in St. Bartholomew's Hospital, which so completely confirms his description of that disease in Dr. Rees's Cyclopædia, and contradicts his suggestions in the Synopsis. As such a case in a public hospital in these northern regions may not occur again, we shall think it our duty to avail ourselves of it, for the benefit of practitioners in the country, who may not have an opportunity of seeing it. We shall be open to every candid remark, and be thankful for information from any source.

---

*Theatre of Anatomy, Medicine, &c. Blenheim-street, Great Marlborough-street.*—Dr. Hooper and Dr. Ager will begin their next Course of Lectures and Examinations at this school, on Monday, February 6, 1815: on the Theory and Practice of Physic, at eight o'clock in the morning; on Chemistry, and the Materia Medica, at nine. Mr. Brookes, on Anatomy, Physiology, and Surgery, will begin on Monday, January 23, at two o'clock.

---

*Medical School of St. Thomas's and Guy's Hospitals.*—The

Spring Course of Lectures at these adjoining Hospitals will commence the beginning of February. viz.

*At St. Thomas's.*—Anatomy and the Operations of Surgery, by Mr. ASTLEY COOPER and Mr. HENRY CLINE.—Principles and Practice of Surgery, by Mr. ASTLEY COOPER.

*At Guy's.*—Practice of Medicine, by Dr. BABINGTON and Dr. CURRY.—Chemistry, by Dr. BABINGTON, Dr. MARCET, and Mr. ALLEN—Experimental Philosophy, by Mr. ALLEN.—Theory of Medicine, and Materia Medica, by Dr. CURRY and Dr. CHOLMELEY.—Midwifery, and Diseases of Women and Children, by Dr. HAIGHTON.—Physiology, or Laws of the Animal Œconomy, by Dr. HAIGHTON.—Structure and Diseases of the Teeth, by Mr. FOX.

---

*Theatre of Anatomy, Bartlett's Court, Holborn.*—Lectures on Anatomy, Physiology, Pathology, and Surgery, by Mr. JOHN TAUNTON, F.A.S. Member of the Royal College of Surgeons of London, Surgeon to the City and Finsbury Dispensaries, City of London Truss Society, &c. The Winter Course will commence on Saturday, January 21, 1815, at eight o'clock in the evening precisely, and be continued every Tuesday, Thursday, and Saturday, at the same hour.

---

Dr. SQUIRE will, on Saturday the 7th of this month, begin a Course of Lectures on the Theory and Practice of Midwifery, and the Diseases of Women and Children.

---

Dr. ADAMS will commence his Spring Course of Lectures on the Institutes and Practice of Medicine, about the end of January.

---

Dr. CLARKE and Mr. CLARKE will begin their next Course of Lectures on Midwifery and the Diseases of Women and Children, on Monday, January 23. The Lectures are read at the house of Mr. CLARKE, 10, Saville Row, Burlington Gardens, every morning, from a quarter past ten to a quarter past eleven, for the convenience of students attending the hospitals.

---

Dr. MERRIMAN'S next Course of Lectures on Midwifery, will commence on the 24th day of January, at the Middlesex Hospital.

---

Dr. CLOUGH, Physician Accoucheur to the St. Mary-le-bone General Dispensary, &c. Berners-street, will commence his usual Course of Lectures on the Science and Practice of Midwifery, including the Diseases of Women and Infants, on Monday morning, January 9th, at half past ten, and in the evening at seven.

N. B. A separate course will be delivered to gentlemen in the army and navy.

---

A Course of Lectures on Electricity and Electro-Chemistry, by Mr. G. J. SINGER, will commence at the Russel Institution, on Monday, January 16, at eight o'clock in the evening, and will be continued on the succeeding Mondays at the same hour.

### LONDON PRICES OF DRUGS.—Dec. 23.

*(To be continued regularly.)*

| | £ | s. | d. | £ | s. | d. | per |
|---|---|---|---|---|---|---|---|
| Aloes Barbadoes, from | 22 | 0 | 0 to | 0 | 0 | 0 | C. |
| ——- Cape | 7 | 10 | 0.. | 8 | 8 | 0 | — |
| ——- Succotrina | 23 | 0 | 0.. | 0 | 0 | 0 | — |
| ——- Epatica or E.I. | 14 | 0 | 0.. | 16 | 0 | 0 | — |
| Angelica Root | 7 | 0 | 0.. | 7 | 5 | 0 | — |
| Alkanet Root | 5 | 10 | 0.. | 6 | 0 | 0 | — |
| Antimony Crude | 2 | 16 | 0.. | 0 | 0 | 0 | — |
| Aqua Fortis | S. | 0 | 8.. | D. | 1 | 1 | lb. |
| Arrow Root fine | 0 | 2 | 0.. | 0 | 3 | 6 | — |
| ——— ordinary | 0 | 0 | 9.. | 0 | 1 | 3 | — |
| Arsenic Red | 5 | 0 | 0.. | 0 | 0 | 0 | C. |
| ——— White | 2 | 12 | 0.. | 2 | 14 | 0 | — |
| Balsam Capivi | 0 | 4 | 9.. | 0 | 5 | 0 | lb. |
| ——— Peru | 1 | 0 | 0.. | 1 | 1 | 0 | — |
| ——— Tolu | 0 | 14 | 6.. | 0 | 15 | 6 | — |
| Bark Jesuits', Com. | 0 | 3 | 6.. | 0 | 4 | 0 | — |
| ——— Second | 0 | 6 | 0.. | 0 | 7 | 0 | — |
| ——— Quil or best | 0 | 8 | 0.. | 0 | 10 | 0 | — |
| ——— Red | 0 | 8 | 8.. | 0 | 9 | 0 | — |
| ——— Yellow | 0 | 2 | 0.. | 0 | 2 | 2 | — |
| Borax refined E.I. | none. | | | | | | |
| ——— English | 0 | 2 | 8.. | 0 | 3 | 0 | lb. |
| ——— unrefined or Tinc. | 9 | 0 | 0.. | 10 | 0 | 0 | C. |
| Camphire refined | 0 | 6 | 0.. | 0 | 6 | 3 | lb. |
| ——— unrefined | 15 | 0 | 0.. | 17 | 0 | 0 | C. |
| Cantharides | 0 | 9 | 0.. | 0 | 10 | 6 | lb. |
| Cardamoms (best) | 0 | 7 | 6.. | 0 | 8 | 0 | — |
| Cassia Buds | 32 | 0 | 0.. | } | 0 | 0 | C. |
| ——— Fistula W.I. | 5 | 5 | 0.. | } in bond | | | — |
| ——— Lignea | 42 | 0 | 0.. | } | 45 | 0 | — |
| Castoreum American | 2 | 10 | 0.. | 3 | 0 | 0 | lb. |
| ——— Russia | 11 | 11 | 0.. | 0 | 0 | 0 | — |
| Castor Oil per bottle 1½ lb. | 0 | 3 | 9.. | 0 | 4 | 3 | bo |
| Cocculus Indicus | 6 | 0 | 0.. | 7 | 0 | 0 | C. |
| Colocynth Turkey | 0 | 5 | 0.. | 0 | 5 | 3 | lb. |
| Columbo Root | 4 | 15 | 0.. | 6 | 0 | 0 | C. |
| Cream of Tartar | 5 | 0 | 0.. | 5 | 5 | 0 | — |
| Gallangal East-India | uncertain | | .. | 0 | 0 | 0 | C. |
| Gentian Root | 3 | 10 | 0.. | 3 | 15 | 0 | — |
| Ginseng | none | | .. | 0 | 0 | 0 | lb. |
| Grains of Guinea | 6 | 10 | 0.. | 0 | 0 | 0 | C. |
| Gum Ammo. Drop | 34 | 0 | 0.. | 0 | 0 | 0 | — |
| ——— Lump | 5 | 0 | 0.. | 7 | 0 | 0 | — |
| ——— Animi | 3 | 10 | 0.. | 7 | 10 | 0 | — |
| ——— Arabic E.I. | 2 | 0 | 0.. | 4 | 0 | 0 | — |
| ——— Turkey Fine | 8 | 15 | 0.. | 9 | 10 | 0 | — |
| ——— Barbary | 5 | 5 | 0.. | 0 | 0 | 0 | — |
| ——— Assafœtida | 10 | 0 | 0.. | 15 | 0 | 0 | — |
| ——— Benjamin | 15 | 0 | 0.. | 60 | 0 | 0 | — |
| ——— Cambogium | 28 | 0 | 0.. | 30 | 0 | 0 | — |
| ——— Copal scraped | 0 | 3 | 0.. | 0 | 4 | 6 | lb. |
| ——— Galbanum | 30 | 0 | 0.. | 0 | 0 | 0 | C. |

| | £ | s. | d. | £ | s. | d. | per |
|---|---|---|---|---|---|---|---|
| Gum Guaiacum, from | 0 | 2 | 6 to | 0 | 3 | 0 | lb. |
| ——— Mastic | 0 | 4 | 6.. | 0 | 4 | 9 | — |
| ——— Myrrh | 15 | 0 | 0.. | 18 | 0 | 0 | C. |
| ——— Olibanum | 8 | 0 | 0.. | 11 | 0 | 0 | — |
| ——— Oppoponax | 80 | 0 | 0.. | 0 | 0 | 0 | — |
| ——— Sandrac | 8 | 0 | 0.. | 8 | 5 | 0 | — |
| ——— Senegal | 6 | 15 | 0.. | 7 | 0 | 0 | — |
| ——— Tragacanth | 28 | 0 | 0.. | 29 | 0 | 0 | — |
| Jalap | 0 | 4 | 9.. | 0 | 5 | 0 | lb. |
| Ipecacuanha | 0 | 14 | 6.. | 0 | 0 | 0 | — |
| Isinglass Book | 0 | 9 | 0.. | 0 | 10 | 0 | — |
| ——— Leaf | 0 | 9 | 0.. | 0 | 10 | 0 | — |
| ——— Long Staple | 0 | 9 | 0.. | 0 | 10 | 6 | — |
| ——— Short Staple | 0 | 9 | 0.. | 0 | 10 | 6 | — |
| Manna Flakey | 0 | 4 | 9.. | 0 | 5 | 0 | — |
| ——— Sicily in Sorts | 0 | 0 | 0.. | 0 | 0 | 0 | — |
| Musk China | 0 | 18 | 0.. | 0 | 0 | 0 | oz. |
| Nux Vomica | 2 | 0 | 0.. | 2 | 2 | 0 | C. |
| Oil of Vitriol | 0 | 0 | 3¾ | 0 | 0 | 4 | lb. |
| Opium East-India | 1 | 3 | .. | 1 | 4 | 0 | — |
| ——— Turkey | 1 | 3 | .. | 0 | 0 | 0 | — |
| Pink Root | 0 | 4 | .. | 0 | 4 | 6 | — |
| Quicksilver | 0 | 5 | 3.. | 0 | 5 | 9 | — |
| Rhubarb East-India | 0 | 9 | 0.. | 0 | 13 | 0 | — |
| ——— Russia | 0 | 18 | 0.. | 1 | 0 | 0 | — |
| Saffron Spanish | 2 | 10 | 0.. | 2 | 12 | 0 | — |
| ——— French | 1 | 10 | 0.. | 2 | 15 | 0 | — |
| Sago | 5 | 5 | 0.. | 6 | 0 | 0 | C. |
| Sal. Ammoniac | 10 | 10 | 0.. | 11 | 0 | 0 | — |
| Sarsaparilla | 0 | 3 | 6.. | 0 | 4 | 6 | lb. |
| Sassafras | 7 | 10 | 0.. | 8 | 0 | 0 | T. |
| Scammony Aleppo | 1 | 9 | 0.. | 1 | 10 | 0 | lb. |
| ——— Smyrna | 0 | 13 | 0.. | 0 | 0 | 0 | — |
| Senna | 0 | 4 | 6.. | 0 | 5 | 0 | — |
| Seeds Anni Alicant | 4 | 15 | 0.. | 5 | 0 | 0 | C. |
| ——— Coriander English | 0 | 14 | 0.. | 0 | 0 | 0 | — |
| ——— Cummin | 5 | 1 | 0.. | 0 | 0 | 0 | — |
| ——— Fenugreek | 1 | 15 | 0.. | 0 | 0 | 0 | — |
| Shellack | 7 | 10 | 0.. | 10 | 15 | 0 | — |
| Sticklack | 3 | 10 | 0.. | 7 | 0 | 0 | — |
| Snake Root | 0 | 15 | 6.. | 0 | 0 | 0 | lb. |
| Soap Castile or Spanish | 9 | 0 | 0.. | 10 | 0 | 0 | C. |
| Spermaceti refined | 0 | 2 | 2.. | 0 | 0 | 0 | lb. |
| Tamarinds West-India | 8 | 10 | 0.. | 9 | 0 | 0 | C. |
| Tapioca Lisbon | 0 | 0 | 9.. | 0 | 1 | 0 | lb. |
| Turmeric Bengal | 4 | 10 | 0.. | 4 | 15 | 0 | C. |
| ——— China | 6 | 10 | 0.. | 7 | 0 | 0 | — |
| ——— West-India | 3 | 10 | 0.. | 4 | 4 | 0 | — |
| Verdigris Wet | 0 | 3 | 6.. | 0 | 0 | 0 | lb. |
| ——— Dry | 0 | 5 | 0.. | 0 | 5 | 6 | — |
| ——— Crystallized | 0 | 8 | 0.. | 0 | 8 | 6 | — |
| Vitriol Roman | 0 | 0 | 7.. | 0 | 0 | 0 | — |
| ——— Foreign white | 2 | 10 | 0.. | 0 | 0 | 0 | C. |

*Price of Vials per Gross.*—8 oz. 70s.—6 oz. 58s.—4 oz. 47s.—3 oz. 43s.—2 oz. 36s.—1 oz. 30s. — ½oz. 24s.

### OBSERVATIONS.

At recent Sales of Merchandize by Auction, Mr. E. Rodwell sold, on the 22d December, 40 sarons Yellow Bark, 2s. to 2s. 2d. per lb.—5 chests Peruvian Bark, 1s. 8d. to 5s. per lb.—5 casks Cream Tartar, 94s. per cwt.—2 casks Tamarinds, 5l. 10s. per cwt.—7 casks Arrow Root, 11d. to 16d. per lb.—1 cask Gum Olibanum, 10d. per lb.

### *Monthly Prices of* SUBSTANCES *used in* PHARMACY,

At Messrs. SELWAY and HENLEY's (Chemical and Pharmaceutical Laboratory,) 35, Upper Mary-le-bone Street, Portland Place.

Acetum Scillæ..........lb. 2*s.* 0*d.*

Abietis Resina ............ 0 10

Acaciæ Gummi, from 2*s.* 3*d.* to 3 6

ACIDUM Aceticum, cong. 3s. 6d.—Benzoic. oz. 6s. 0d.—Oxalic. lb. 18s. 0d. —Nitric. 3s. 9d.—Nitros. 2s. 8d.—Muriatic. 0s. 9d.—Sulphuric. 0s. 6d. —Sulph. Arom. 5s 0d.

Alcohol sp. gr. 815 M. lb. 5 0

Æther sulphur. sp. gr. 789 10 6

——— rectificatus sp. gr. 744 12 0

Ærugo ..............lb. 6 6

Aloes spicatæ extractum .... 5 0

Alumen, 4*d.* exsiccatum.... 0 8

Ammoniæ Murias, 2*s.* 2*d.* to 2 9

——— Carbonas ........ 3 6

Amyg. Amaræ, 1*s.* 6*d.* Dulc. 5 0

Ammoniacum, from 6*s.* 6*d.* to 12 6

Anthemidis Flores, 1*s.* 9*d.* to 2 0

Antimonii Sulphuretum .... 1 0

——— Aurum. Sulph. .. 6 6

——— Murias ........ 3 9

Antimonium Tartarizatum .. 5 0

Aqua Cinnamomi vera .... 18 0

—— Rosæ .............. 5 6

Aliæ Aquæ, from 2*s.* 6*d.* to 3 6

Arsenici Oxydum........lb. 1 0

Argenti Nitras ............ 6 9

Assafœtida Gummi-resina .. 6 9

Aurantii Cortex ........ 2 6

Balsamum Peruvianum...... 33 0

——— Tolutanum ...... 15 0

Benzoinum elect. .......... 11 0

Calamina præp. .......... 0 3

Calumbæ Radix .......... 2 6

Cambogia opt. ............ 9 6

Camphora ................ 7 3

Canellæ Cortex .......... 3 9

Cardamomi Semina opt..... 12 0

Cascarillæ Cortex .......... 6 0

Castoreum ............unc. 4 4

Caryophilli ............ lb. 11 6

Cassiæ Cortex ............ 8 0

Catechu Extractum ........ 2 3

Cetaceum ................ 3 0

Cera flava, 3*s.* 6*d.* alba .... 3 9

Cerata, from 2*s.* 3*d.* to .... 4 8

Cinchonæ cordif. Cort....... 5 6

——— lancif. Cort....... 13 0

——— oblongif. Cort..... 16 0

Cinnamomi Cortex ........ 16 0

Coccus (Coccinella) ........ 64 0

Colocynthidis ............ 7 6

Copaiba.................. 6 6

Cretæ ppt. .............. 0 8

Croci stigmata ............ 64 0

Cupri sulphas ............ 1 0

Cuprum ammoniatum ...... 8 0

Curcumæ Pulvis .......... 2 6

CONFECTIO Aromatica, 8s. 6d.—Aurantii, 2s. 9d.—Opii, 4s. 6d.—Rosæ Gallicæ, 1s. 10d.—RosæCaninæ, 1s. 8d. —Scammoniæ, 15s. 0d.—Sennæ, 2s. —Amygdalæ, 5s. 0d.

EMPLASTRA Ammon. 8s. 6d.—Cumini, 2s.—Galbani Comp. 1s. 8d.—Lyttæ, 6s. 6d.—Opii, 2s. 6d.—Plumbi, 1s. 6d. —Resinæ, 1s. 6d.—Roborans, 2s. 0d. —Saponis, 2s. 6d.—Hydrarg. 2s. 6d. —Hydrar. cum Amm. 6s. 0d.

EXTRACTA Anthemidis, unc. 1s. 0d. —Belladon. 1s. 3d.—Cinch. 2s 3d.—Cinch. resinos. 4s. 0d.—Coloc. 2s. 0d. —Coloc. comp. 0s. 10d.—Conii, 0s. 6d. —Gent. 0s. 5d.—Glycyr. lb. 3s 0d.—Cascar. unc. 3s. 0d.—Hæmatoxyli, 0s. 5d.—Humuli, 0s. 9d.—Hyoscyami, 1s. 0d.—Jalapæ, 1s. 6d. Res. 2s. 8d. —Opii aquos, 3s. 8d.—Opii resinos, 3s. 0d.—Papaveris, 0s. 6d.—Rhæi, 2s. 4d.—Sarsapar. 10d.—Taraxaci, 6d.

Ferri carbon. 1*s.* 0*d.* sulph. lb. 1 0

Ferrum Ammoniatum ...... 3 6

——— Tartarizatum ...... 2 4

Galbani Gummi-resinæ .... 8 6

Gentianæ Radix ........ 1 3

Guaiaci Resina ............ 7 6

Hydrargyrus purificatus .... 5 4

——— cm Creta ...... 4 0

Hydrargyri Oxymurias...... 8 0

——— Submurias ...... 8 6

——— Hydros. 10 0

——— Nitrico-Oxydum .. 8 6

——— Oxydum Rubrum 5 0

——— Sulphuretum Nig. 3 6

——— Præcipitatus Alb. 8 0

Hellebori nigri Radix ...... 2 6

Ipecacuanhæ Radix ........ 18 0

——— Compos. Pulv. lb. 8 0

Jalapæ Radix.............. 6 6

Kino .................... 12 0

Liquor Plumbi Acetatis M. lb. 1 0

——— Ammoniæ .... P.L. 2 8

——— Vol. Corn. Cerv. .... 1 3

Linimentum Camphoræ comp. 5 0

——— Saponis comp. .. 5 0

Lichen Islandicus......P. lb. 1 9

Lini Semina .............. 0 6

Lyttæ .................. 11 0

Magnesia ................ 8 0

——— Carbonas ........ 3 0

——— Sulphas ........ 1 2

Manna optima ............ 6 6

Mel Rosæ................ 2 6

—— Despumat.............. 3 0

Moschus pod, 24*s.* in gr. unc. 40 0

Mastiche .............. lb. 5 6

Myristicæ Nuclei.......... 25 0

Myrrha elect. ............ 6 6

Opium (Turkey) ........ 34 0

—— (East Inndia)......lb. 32 0

Oleum Amygdalæ ...... lb. 3 9

| | | |
|---|---|---|
| Oleum Anisi ........... unc. | 2 | 0 |
| —— Anthemidis .......... | 5 | 0 |
| —— Cinnamomi Cassiæ .... | 8 | 0 |
| —— Caryophili .......... | 5 | 0 |
| —— Cajuputi ............ | 8 | 0 |
| —— Carui ............... | 1 | 3 |
| —— Juniperi ............ | 0 | 9 |
| —— Lavandulæ ........... | 5 | 0 |
| —— Lini .......... cong. | 6 | 0 |
| —— Menthæ piperitæ unc. | 3 | 6 |
| ———— viridis ...... | 3 | 6 |
| —— Olivæ, from 12*s*. 0*d*. to | 18 | 0 |
| —— Origani .......... unc. | 2 | 0 |
| —— Pimentæ ............. | 4 | 6 |
| —— Ricini, from 7*s*. 0*d*. to | 10 | 0 |
| —— Rosmarini ...... unc. | 0 | 9 |
| —— Succini .. 2*s*. 4*d*. rect. | 2 | 6 |
| —— Sulphuretum ...... lb. | 1 | 6 |
| —— Terebinthinæ rectif. .. | 2 | 0 |
| Oxymel Scillæ ............ | 3 | 0 |
| Papaveris Capsulæ (per 100) | 2 | 3 |
| Plumbi Carbonas ........ lb. | 0 | 8 |
| —— Superacetas ........ | 2 | 2 |
| Potassa Fusa ........... unc. | 0 | 6 |
| —— cum Calce ........ | 1 | 6 |

Potassæ—Nitras, lb. 1s.—Acetas, 8s. —Subcarbon. 1s. 2d.—Carbon. 4s 6d. —Tartras, 3s.—Supertartras, 1s. 10d.

| | | |
|---|---|---|
| Pilulæ Hydrargyri ........ | 6 | 0 |
| —— Aloes comp. ........ | 8 | 0 |
| ———— cum Colocynth. | 16 | 0 |
| —— Scillæ Compos. .... | 6 | 6 |
| —— e Styrace .......... | 48 | 0 |
| Piper longum et Nigrum .. | 2 | 6 |
| Pimento .............. lb. | 2 | 6 |
| Pulvis Aloes cum Canella .. | 6 | 6 |
| ———— Compositus .... | 10 | 0 |
| —— Antimonialis ........ | 7 | 0 |
| —— Cinnamomi Comp. .. | 16 | 0 |
| —— Cornu cervi cum Opio | 14 | 0 |
| —— Cretæ Composit. .... | 8 | 0 |
| ———— cum Opio | 8 | 0 |
| —— Kino Compositus .... | 12 | 0 |
| —— Scammoniæ Comp. unc. | 2 | 6 |
| —— Sennæ Composit. .. lb. | 8 | 0 |
| —— Tragacanthæ Comp. .. | 5 | 0 |
| Resina Flava et Nigra ...... | 0 | 5 |
| Rosæ Folia .............. | 8 | 0 |
| Ras Quassiæ .............. | 1 | 6 |
| Rhæi Radix (Russia) ...... | 32 | 0 |
| ———— (East India) .... | 14 | 0 |
| Sapo (Spanish) ............ | 2 | 6 |
| Sarsaparilla Radix Inc. ...... | 5 | 0 |
| Scammoniæ Gum-Resina unc. | 2 | 6 |
| Scilla recens, 1*s*. 3*d*. siccata .. | 3 | 6 |
| Sennæ Folia Opt. Alexand. .. | 6 | 6 |
| Simaroubæ Cortex ........ | 4 | 0 |
| Sinapis super. ............ | 2 | 6 |
| —— secund. ............ | 2 | 0 |
| Sodæ Boras .............. | 4 | 0 |
| —— Carbonas ............ | 7 | 0 |
| —— Phosphas ............ | 2 | 8 |
| —— Subcarbonas .......... | 1 | 4 |
| —— Sulphas ............ | 0 | 8 |
| Soda tartarizata ........ lb. | 2 | 4 |
| Spongia usta ........... unc. | 1 | 3 |
| Spiritus Ammoniæ .... M. lb. | 3 | 6 |
| ———— aromaticus | 4 | 6 |
| ———— fœtidus .. | 3 | 6 |
| ———— succinatus | 4 | 6 |
| —— Cinnamomi ver. .... | 3 | 6 |
| —— Juniperi Compos. .. | 3 | 0 |
| —— Lavandulæ ........ | 4 | 6 |
| ———— Compos. | 5 | 0 |
| —— Myristicæ ........ | 3 | 0 |
| —— Pimentæ .......... | 3 | 0 |
| —— Rosmarini ........ | 3 | 0 |
| —— Ætheris Aromaticus | 6 | 0 |
| ———— Compositus | 7 | 0 |
| —— Nitrici sp. gr. 860 | 5 | 6 |
| ———— Sulphurici | 6 | 0 |
| —— Vini rectificatus .... | 26 | 0 |
| Syrupi, from 1*s*. 8*d*. to .... | 3 | 0 |
| Sulphur .................. | 0 | 6 |
| —— Præcipitatum ...... | 1 | 0 |
| Tamarindus opt. .......... | 2 | 3 |
| Terebinthina Vulgaris ...... | 0 | 9 |
| ———— Canadensis .... | 8 | 0 |
| ———— Chia .......... | 6 | 0 |
| Tragacantha Gummi ........ | 7 | 0 |

Tincturæ—Aurantii, M. lb. 3s. 9d.—Aloes, 4s. 0d.—Aloes Comp. 9s. 6d.—Assafœt. 5s 6d.—Balsa. Tolut. 5s. 6d. —Benzoini Comp. 6s. 9d.—Camph. Comp. 4s. 0d.—Cardam. 4s. 0d.—Cardam. Comp. 4s. 0d.—Calum. 3s. 9d. Capsici, 3s. 9d.—Cascar. 4s 6d.—Cast. 7s. 0d.—Catechu, 4s. 0d —Cinchonæ, 6s. 0d —Cinch. Comp. 6s. 0d.—Cinch. Am. 7s. 0d.—Croci, 6s. 6d.—Digitalis, 4s. 6d.—Ferri Ammon. 4s. 0d.—Cinnam. 4s. 6d.—Cin. Comp. 4s. 6d. Gent. Comp. 4s. 0d.—Guaiaci, 6s. 0d. —Guaiaci Am. 6s. 0d.—Helleb. Nig. 4s. 6d.—Humuli, 5s. 0d.—Hyoscyami Nig. 4s. 6d.—Jalap. 4s. 6d.—Japon, 4s. 6d.—Fer. Mur. 5s. 0d.—Kino, 5s. 0d.—Lyttæ, 3s. 9d.—Myrrhæ, 4s. 6d.—Opii, 7s. 0d.—Opii Camph. 4s. 6d.—Quassiæ, 3s. 9d.—Rhei, 4s. 6d. —Rhei Comp. 4s. 0d.—Scillæ, 4s. 6d. —Sennæ, 4s. 9d.—Serpent. 6s. [illegible]d.—Valer. 4s. 6d.—Valer. Am. 5s. 6[illegible]—Zingiberis, 4s. 6d.

| | | |
|---|---|---|
| Valerianæ Radix .......... | 1 | 8 |
| Veratri Radix ............ | 1 | 6 |

Unguenta—Hydrar. fort, 5s. 0d.—Hydrar. Mit. 3s. 0d.—Hydrar. Nitratis, 3s. 3d.—Hydrar. Nitrico-oxy. 2s. 9d. —Sulph. Comp. 1s. 9d.—Alia ung. 1s. 8d. to 3s. 6d.

| | | |
|---|---|---|
| Uvæ Ursi Folia ............ | 2 | 4 |

Vina—Aloes, 3s. 6d.—Antimoniale, 3s. 0d.—Ferri, 3s. 6d.—Ipecac. 5s. 6d. —Opii, 8s. 0d.

| | | |
|---|---|---|
| Zinci Oxydum ............ | 4 | 6 |
| —— Sulphas purif. ...... | 2 | 0 |
| Zingiberis Radix opt. .... | 3 | 2 |

Leeches, 24s. per hundred.—Essential Salt of Lemons, 4s. 6d. per dozen.

### METEOROLOGICAL REGISTER.

*From November the 25th, to December the 25th,* 1814.

Kept by C. BLUNT, Philosophical Instrument Maker, No. 38, Tavistock-Street, Covent-Garden.

| Moon. | Day. | Wind. | Barometrical Pressure. Max. | Min. | Mean. | Temperature. Max. | Min. | Mean. | |
|---|---|---|---|---|---|---|---|---|---|
| | 26 | W | 29·46 | 29·22 | 29·305 | 50 | 34 | 44·3 | Rain |
| ○ | 27 | W | 29·5 | 29·5 | 29·5 | 45 | 36 | 41·6 | Rain |
| | 28 | N | 29·73 | 29·67 | 29·697 | 48 | 35 | 40·6 | Rain |
| | 29 | W | 29·8 | 29 33 | 29·527 | 48 | 38 | 41·6 | Rain |
| | 30 | SW | 29·28 | 29·19 | 29 217 | 44 | 36 | 40·3 | Fair |
| | 1 | NW | 29·66 | 29·45 | 29·543 | 45 | 37 | 40·6 | Fair |
| | 2 | N | 29·80 | 29·74 | 29·772 | 46 | 35 | 39 | Cloudy |
| | 3 | NW | 29 88 | 29·88 | 29·88 | 47 | 36 | 39·6 | Nights } Foggy |
| ◑ | 4 | N | 29·64 | 29·64 | 29·64 | 50 | 34 | 39·6 | Foggy |
| | 5 | NW | 29·59 | 29·42 | 29·576 | 46 | 33 | 41 | Foggy |
| | 6 | N | 30·08 | 29·9 | 30· | 46 | 32 | 38 | Clear |
| | 7 | W | 29·80 | 29·69 | 29·745 | 46 | 39 | 40 | Clear |
| | 8 | NW | 29·63 | 29·45 | 29·51 | 49 | 33 | 43 | Rain |
| | 9 | NE | 29·54 | 29·32 | 29·452 | 53 | 31 | 42·3 | Rain |
| | 10 | N | 29·69 | 29 32 | 29 45 | 52 | 35 | 40·3 | Fair |
| ● | 11 | W | 29·47 | 29·47 | 29·47 | 53 | 50 | 49·9 | Rain |
| | 12 | SW | 29·79 | 29 68 | 29·722 | 57 | 53 | 55·3 | Rain Hard Gale |
| | 13 | SW | 29·55 | 29·52 | 29 53 | 58 | 42 | 52 | Rain Hard Gale |
| | 14 | NW | 29·73 | 29·64 | 29 67 | 56 | 45 | 49·3 | Gale Fair |
| | 15 | SW | 29.65 | 29·55 | 29 6 | 57 | 41 | 50·6 | Fair Gale |
| | 16 | SW | 29·92 | 29·43 | 29·612 | 55 | 40 | 50·3 | Rain Gale |
| | 17 | SW | 29·92 | 29·73 | 29 79 | 57 | 45 | 47 | Rain Gale |
| | 18 | W | 29·70 | 29·70 | 29·70 | 59 | 50 | 54·3 | Rain |
| ◐ | 19 | NW | 29·83 | 29 67 | 29·712 | 55 | 32 | 47·6 | Fair |
| | 20 | N | 30·04 | 30·01 | 30·026 | 40 | 31 | 36 | Fair |
| | 21 | NE | 29·97 | 29·89 | 29·925 | 37 | 30 | 34 | Fair Lunar Halo this Evening |
| | 22 | NE | 29·69 | 29·60 | 29·637 | 44 | 31 | 35·6 | Fair |
| | 23 | NE | 29·60 | 29·49 | 29·567 | 41 | 30 | 35·6 | Fair |
| | 24 | E | 29·65 | 29·61 | 29·63 | 36 | 30 | 32·3 | Snow |
| | 25 | E | 29·60 | 29 60 | 29·60 | 37 | 31 | 33 | Snow |

RESULTS.

| | | | | | |
|---|---|---|---|---|---|
| Mean barometrical pressure of the month | | 29·633 | Mean temperature of the month | | 42·486 deg. |
| Maximum 30·08 | wind at | N | Maximum 59, | wind at | W |
| Minimum 29·19 | —— | SW | Minimum 30, | —— | NE |

*Scale exhibiting the prevailing Winds during the Month.*

| N | NE | E | SE | S | SW | W | NW |
|---|---|---|---|---|---|---|---|
| 6 | 4 | 2 | 0 | 0 | 6 | 6 | 6 |

| | Mean barometrical pressure. | Mean temperature. |
|---|---|---|
| From the full moon on the 27th Nov. to the last quarter on the 4th Dec. | 29·59 | 40·47 |
| —— last quarter on the 4th, to the new moon on the 11th | 29·624 | 40 6 |
| —— new moon on the 11th, to the first quarter on the 19th | 29·636 | 51·08 |
| —— first quarter on the 19th, to the full moon on the 26th | 29·727 | 36·3 |

